# Elevated Serum Bisphenol A Level in Patients with Dilated Cardiomyopathy

**DOI:** 10.3390/ijerph120505329

**Published:** 2015-05-19

**Authors:** Qinmei Xiong, Xiao Liu, Yang Shen, Peng Yu, Sisi Chen, Jinzhu Hu, Jianhua Yu, Juxiang Li, Hong-Sheng Wang, Xiaoshu Cheng, Kui Hong

**Affiliations:** 1 Department of Cardiovascular, The Second Affiliated Hospital of Nanchang University, Nanchang 330000, China; E-Mails: xiongqinmei@gmail.com (Q.X.); kellyclarkwei@163.com (X.L.); sain.yong@gmail.com (Y.S.); yupeng_jxndefy@163.com (P.Y.); cheinsee@163.com (S.C.); hujinzhu1983@sina.com (J.H.); yujianhua@medmail.com.cn (J.Y.); ljx912@126.com (J.L.); xiaoshumenfan@126.com (X.C.); 2 Department of Pharmacology, College of Medicine, University of Cincinnati, Cincinnati, OH 2301, USA; E-Mail: wanghs@uc.edu; 3 The Key Laboratory of Molecular Medicine, The Second Affiliated Hospital of Nanchang University, Nanchang 330000, China

**Keywords:** Bisphenol A, dilated cardiomyopathy, sex hormone, sex hormone-binding globulin

## Abstract

Background: This study aimed to determine serum Bisphenol A (BPA) concentrations in patients with dilated cardiomyopathy (DCM) as well as the association between serum BPA and several hormonal parameters in DCM patients compared with a healthy control group. Materials and methods: Eighty-eight DCM patients and 88 age- and gender-matched healthy controls were included. Serum BPA levels and several hormonal parameters (including total testosterone (T), sex hormone-binding globulin (SHBG) and estradiol (E2) were measured by using corresponding ELISA Kits. The free androgen index (FAI) was calculated by the formula: total T in nmol/L × 100/SHBG in nmol/L. Results: BPA levels in the total DCM group were significantly higher compared with that in the controls (6.9 ± 2.7 ng/mL *vs.* 3.8 ± 1.9 ng/mL, *p* < 0.001). Significant difference was also observed in SHBG and FAI between DCM patients and controls, (76.9 ± 30.9 nM/L *vs.* 41.0 ± 15.6 nM/L and 2.9 ± 3.5 *vs.* 5.3 ± 2.6, respectively, both of *p* < 0.001). Similar trends were observed in the male and female subgroup. Mean T level was lower in DCM group than in control group (540.8 ± 186.0 pg/mL *vs.* 656.3 ± 112.9 pg/mL, *p* < 0.001). Linear regression analysis has shown that increasing serum BPA levels were statistically significantly associated with increased SHBG levels. However, no statistical difference was noted for E2. Conclusion: Our findings firstly demonstrated that BPA exposure increased in DCM patients compared with that in healthy controls, while FAI and T levels decreased. SHBG presented a positive association with BPA. It is concluded that hormone disorder induced by BPA exposure might be an environmental factor in the pathology of DCM.

## 1. Introduction

Bisphenol A (BPA) has become one of the highest-production-volume chemicals worldwide [1]. It consists in numerous consumer products, such as baby bottles, food and drink packaging, and dental materials, *etc.* Once these plastic consumer products are exposed to high temperatures, acidic or basic solutions, BPA can leach into the environment, water or even food products [2]. Thus, BPA can be detected in a variety of human tissues and body fluid [3]. Previous study of the general United States (U.S.) population has documented that over 90% of the research cohort had detectable BPA in their urine [4].

Recent epidemiological studies have raised concerns regarding the possible association between higher BPA exposure and an increased risk for several cardiovascular diseases. Data from the 2003–2004 National Health and Nutrition Examination Survey (NHANES) provided the first cross-sectional survey on the association between high urinary BPA concentration and increased risk for chronic medical disorders such as cardiovascular disease, diabetes, and abnormal liver function [5]. Higher urinary BPA levels were found in participants who were diagnosed with coronary artery disease, hypertension, angina or heart attack, and reduced heart rate variability [6,7,8,9].

Dilated cardiomyopathy (DCM) is characterized by ventricular chamber enlargement and systolic dysfunction with normal left ventricular wall thickness. The age-adjusted incidence rate between male and female is estimated to be 3:1 for DCM [10]. Although the exact reason for the sex difference remains unclear, the potential mechanisms may involve the sex hormones which may have an effect on cardiac structure and function. BPA is considered to be an endocrine-disrupting chemical which is ubiquitous in human living environments. Both estrogenic and anti-androgenic activities of BPA have been reported, as well as its reproductive toxicity [11]. On the basis of available evidence from epidemiological and experimental studies [12,13], we conducted a case control study including DCM patients and healthy controls to examine the serum levels of BPA and some sex hormones among all participants to evaluate the possible association between BPA exposure and DCM.

## 2. Materials and Methods

All subjects gave their informed consent for inclusion before they participated in the study. The study was conducted in accordance with the Declaration of Helsinki, and the protocol was approved by the Ethics Committee of the Second Affiliated Hospital of Nanchang University (Ethical code: No. 2012092).

### 2.1. Study Population

Eighty-eight patients diagnosed with DCM were included from the cardiovascular department in the Second Affiliated Hospital of Nanchang University from March 2012 to July 2013. The diagnostic criterion of DCM referred to the American Heart Association Scientific Statement from the Council on Clinical Cardiology [14]. It is mainly based on symptoms and ventricular chamber enlargement plus systolic dysfunction with normal left ventricular wall thickness documented by echocardiography. The control group was comprised of 88 age- and gender-matched healthy volunteers from the medical center of the Second Affiliated Hospital of Nanchang University during the same period. Subjects with any of the following conditions will be excluded from the study: taking hormonal drugs in the last 3 months, overt renal and/or hepatic impairment, or overt endocrinology disorder.

### 2.2. Sample Collection

Venous fasting blood was drawn in the morning and collected into a general vacuum tube. The samples were centrifuged after retraction of the clot. Then the serum was separated and stored at −80 °C until assayed for BPA, total testosterone (T), sex hormone-binding globulin (SHBG) and estradiol (E2).

### 2.3. Sex Hormone Measurement

Serum total T, SHBG and E2 were measured by enzyme-linked immunosorbent assay (ELISA) by using commercially available ELISA kits (Uscn Life Science, Wuhan, China) accordingly. Detailed methods for all assays referred to the manufacturer’s instructions. All standards and samples were measured in duplicate. The free androgen index (FAI) was calculated by the formula: total T in nmol/L × 100/SHBG in nmol/L.

### 2.4. BPA Elisa Measurement

The serum concentrations of BPA were measured with a commercially available ELISA kit (Code No. 27766, IBL Co., Ltd., Gunma, Japan) which is capable of the quantitative determination of BPA levels in human serum or plasma samples. The IBL’s assay kit is based on competitive ELISA protocol by using anti-rabbit IgG antibody coated solid-phase method. It has a measurement range of 0.3–100 ng/mL BPA. All experimental procedures were carried out in accordance with the manufacturer’s instructions. Duplicate measurements of all test samples and standard were performed. According to the manufacturer’s instructions, the ranges of the intra and inter-assay coefficients of variation were 5.5–14.0 and 4.3%–5.2%, respectively.

### 2.5. Statistical Analyses

Data analysis was performed by using the statistical package SPSS version 18.0 (SPSS, Inc., Chicago, IL, USA). Continuous variables were examined for normal distributions and expressed as mean ±SD, and compared between the two groups by using Student’s t-test or Mann-Whitney test as a function of the normalcy of the data. Categorical variables were compared by using the Chi-square test. Multiple linear regression analysis was used to investigate the association between serum BPA levels and hormonal parameters after controlling and adjusting for potential confounders. All tests are two-sided, and a P value less than 0.05 was considered statistically significant.

## 3. Results

The demographic characteristics of all participants were shown in Table 1. There were 59 men *vs.* 29 women with DCM, and the male *vs.* female ratio of DCM was 2:1. The mean age of DCM group was 59.6 ± 13.2 years, while the mean age of healthy controls was 59.0±12.7 years. No statistical difference between the two groups was noted as a function of age and gender.

**Table 1:** Demographic characteristics of two groups

Variable	DCM (n = 88)	Control (n = 88)	*p* Value
Age, years	59.6 ± 13.2	59.0 ± 12.7	0.767
Male, n (%)	59 (67%)	55 (63%)	0.318

Summary statistics for serum BPA levels and hormone parameters of DCM patients and healthy controls were shown in Table 2. Serum BPA levels were significantly higher in DCM group compared with that in controls (6.9 ± 2.7 *vs.* 3.8 ± 1.9 ng/mL, *p* < 0.001). Lower serum T levels and higher SHBG levels in DCM group compared with controls (488.3 ± 188.2 *vs.* 555.8 ± 165.8 pg/mL, 76.9 ± 30.9 *vs.* 41.0 ± 15.6 nM/L, respectively), while FAI was significantly lower in DCM patients (2.9 ± 3.5 *vs.* 5.3 ± 2.6, *p* < 0.001).

**Table 2:** Results in the total group, as well as in male and female subgroups.

Variables	Total (Mean ± SD)		Male (Mean ± SD)		Female (Mean ± SD)	
	DCM (n = 88)	Controls(n = 88)	DCM (n = 59)	Controls(n = 55)	DCM (n = 29)	Controls (n = 33)
Age (years)	59.6 ± 13.2	59.0 ± 12.7	58.0 ± 14.3	56.7 ± 14.0	62.7 ± 10.4	62.8 ± 9.2
BPA (ng/mL)	6.9 ± 2.7 **	3.8 ± 1.9 **	7.0 ± 2.9 **	3.9 ± 2.0 **	6.5 ± 2.2 **	3.8 ± 1.8 **
E2 (pg/mL)	17.7 ± 10.5	18.1 ± 9.7	17.8 ± 12.0	18.0 ± 11.7	17.6 ± 7.0	18.3 ± 5.2
T (pg/mL)	488.3 ± 188.2 *	555.8 ± 165.8 *	540.8 ± 186.0 **	656.3 ± 112.9 **	381.7 ± 144.1	388.4 ± 83.1
SHBG (nM/L)	76.9 ± 30.9 **	41.0 ± 15.6 **	75.4 ± 30.8 **	42.2 ± 17.8 **	80.0 ± 31.2 **	39.1 ± 11.1 **
FAI	2.9 ± 3.5 **	5.3 ± 2.6 **	3.5 ± 4.2 **	6.2 ± 2.6 **	1.9 ± 0.8 **	3.8 ± 1.8 **

* *p* < 0.05; ** *p* < 0.001; between DCM and controls.

**Figure 1:**
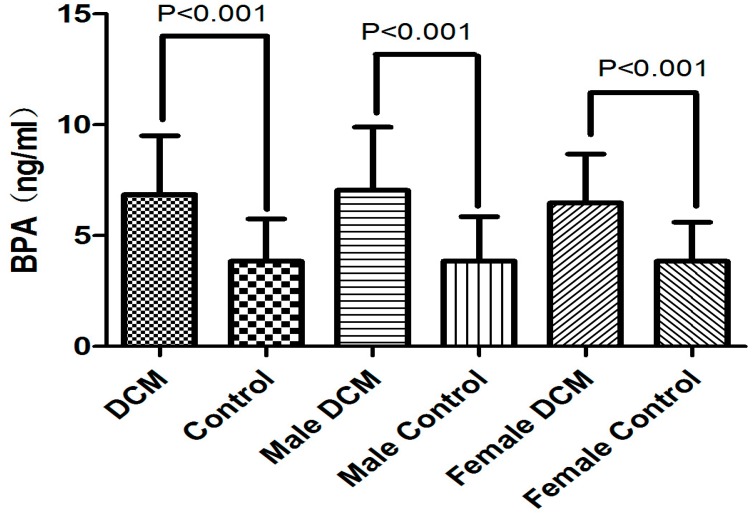
Serum BPA levels in DCM patients and controls, as well as in male and female subgroups.

Both DCM patients and healthy controls were divided into two gender subgroups. DCM males as well as DCM females were comparable for age with their corresponding control groups, respectively. BPA values were significantly higher in DCM male group compared with that in male controls (7.0 ± 2.9 *vs.* 3.9 ± 2.0 ng/mL, *p* < 0.001) and the same difference was noted in the female subgroups (6.5 ± 2.2 *vs.* 3.8 ± 1.8 ng/mL, *p* < 0.001) (Figure 1). Significantly higher serum SHBG levels but lower FAI values were observed in both males and females with DCM (*p* < 0.001). There was significant difference in serum T levels between DCM males and the corresponding controls, but no difference was observed in the female subgroup. Additionally, E2 level presented no statistical difference between DCM patients and healthy controls.

To assess the association between serum BPA level and hormone parameters, we conducted a linear regression analysis among all participants. There was only a statistically significant association between serum BPA and SHBG (β = 0.041; 95% CI, 0.024–0.058, *p* < 0001) (Table 3).

**Table 3:** Linear regression analysis for independent valuables and BPA concentrations.

Independent Valuables	β (95% CI)	*p* Value
Age (years)	0.110(−0.21–0.043)	0.514
E2 (pg/mL)	−0.007(−0.048–0.034)	0.743
T (pg/mL)	−0.002(−0.004–0.001)	0.230
SHBG (nmol/L)	0.041(0.024–0.058)	0.000 **
FAI	0.126(−0.054–0.306)	0.168

** *p* < 0.001.

## 4. Discussion

Even though the genetic, physiological and pathophysiological contributors to DCM have been extensively investigated, much less is known about the latent effect of environmental substances, such as BPA. In this study, our findings firstly demonstrate that serum BPA levels are significantly higher in DCM patients in comparison to healthy controls.

BPA exposure has been linked by various studies throughout the world with a whole host of human health problems, mainly including reproductive toxicity, breast cancer, metabolic syndrome, obesity, non-insulin dependent diabetes mellitus, and cardiovascular disorders (e.g., hypertension, heart rate variability and the severity of atherosclerosis)[6,7,8,9,11]. A series of experimental studies have elucidated the underlying molecular mechanisms of the potential BPA toxicity on the cardiovascular system [15,16,17,18]. These findings have documented that BPA exposure at environmental relevant low-dose may serve as an auxiliary cause for promoting arrhythmia and influencing cardiac structure or function. In line with this evidence, higher BPA exposure could be significantly associated with arrhythmia and heart failure, both of which are the most common clinical manifestations during the pathophysiological development of DCM. Additionally, a recent experimental study has investigated the effects of long-term exposure to BPA on the rat myocardium. Their results demonstrate that long-term BPA exposure induces cardiomyopathy in male rats by impairing mitochondrial function and disturbing methylation of PGC-1α [19].

In the present study, we observed serum BPA concentration rather than urine sample in view that serum BPA concentration more likely reflects the chronic exposure level. Available data from previous biomonitoring studies clearly illustrate that the general population is at risk of unconjugated BPA exposure, which can be routinely detected in blood [20]. Since our study subordinately aimed to assess the association between BPA level and serum hormone levels, serum BPA levels may be a better biomarker compared with urine BPA levels. Additionally, our study showed serum BPA level in the range of 0.16–15.3 ng/mL in all participants, with 99.5% above the current limit of detection of 0.39 bng/mL. However, a lower detection rate of serum BPA level was reported from 20 individuals [3]. Nevertheless, concerning the possible reasons for this deviation, it may be explained by the fact that varying detection methods and the specific populations studied.

To our knowledge, previous study has indicated that the incidence rate of DCM is higher in men than in women [10]. However, the exact reason for the gender-specific discrepancy has yet to be elucidated. In light of this point, we examined the hormonal parameters for all participants to identify the potential correlation between BPA and sex hormones. Our data demonstrate lower T and FAI level but higher SHBG and BPA level in DCM patients compared with those in controls. With regards to a lower T and FAI, BPA has been considered to have an anti-androgenic activity, although it is also found to be a strong SHBG ligand. SHBG level has not been detected in DCM patients previously. However, a potential relationship between SHBG and cardiovascular disease can be found according to a study from Pascual-Figal *et al.*, which demonstrated that SHBG level was associated with the severity of heart failure and a higher risk of cardiac death [21].

Additionally, our data also disclosed a positive association between BPA and SHBG, which was consistent with a handful of previous studies in the male population [22,23,24]. A significant positive association between the BPA and SHBG was detected from urine among 75 men [22]. It has been speculated that androgen action lowers serum SHBG whereas estrogen action increases it; consequently, the estrogenic action of BPA may directly lead to the increase in SHBG levels. These findings may not only provide insights into the impact of BPA on the sex hormonal parameters, but also allow for speculation for the potential cause of the gender difference.

The main limitation of our study is the relatively small sample size, which makes it unreasonable to extrapolate these findings to the general DCM population. Further study with larger sample sizes or basic research is necessary to verify the underlying link between BPA exposure and DCM pathogenesis. In addition, some basic characteristic data were unavailable for part-time participants to perform further analysis. Nevertheless, both DCM patients and healthy controls were age- and gender-matched to ensure comparability.

## 5. Conclusion

Our findings demonstrated that serum BPA level is higher in DCM patients than in healthy controls. This environmental endocrine-disrupting compound may be associated with the gender disparity in the incidence of DCM. A positive association between BPA and SHBG was found which is consistent with previous epidemiologic findings. If these results were confirmed in future larger populations or registry studies, further analysis is definitely warranted to identify the specific mechanism underlying the association between higher BPA exposure and DCM.
